# Supplementary material for: Early brain microstructural development among preterm infants requiring caesarean section versus those delivered vaginally
Source: Sci Rep. 2023 Dec 6;13:21514. doi: 10.1038/s41598-023-48963-z (PMC10700578; doi:10.1038/s41598-023-48963-z)
Supplement: Supplementary file 1 — Supplementary Information. [file 41598_2023_48963_MOESM1_ESM.docx]

**Supplemental Tables**

Supplemental Table 1. Tract-specific linear regression analysis of the association of delivery method with fractional anisotropy values after controlling for gestational age at birth, gestational age at scan, 5-minute APGAR, birth weight Z-score, presence of preeclampsia, and presence of chorioamnionitis as covariates.

| **Tract** | **Coefficient (95% Confidence Interval)** | **Original p-value** | **Corrected p-value** |
| --- | --- | --- | --- |
| Middle cerebellar peduncle | -4.60E-03(-1.50E-02 to 5.79E-03) | 0.38 | 0.52 |
| Pontine crossing tract | -5.15E-03(-2.23E-02 to 1.20E-02) | 0.55 | 0.63 |
| Genu of corpus callosum | -1.40E-02(-2.58E-02 to -2.29E-03) | **0.02** | 0.19 |
| Body of corpus callosum | -1.66E-02(-2.82E-02 to -5.05E-03) | **0.01** | 0.13 |
| Splenium of corpus callosum | -9.84E-03(-2.13E-02 to 1.60E-03) | 0.09 | 0.33 |
| Fornix | -1.26E-02(-3.17E-02 to 6.38E-03) | 0.19 | 0.41 |
| Corticospinal tract R | 5.78E-04(-1.30E-02 to 1.42E-02) | 0.93 | 0.97 |
| Corticospinal tract L | -1.06E-02(-2.29E-02 to 1.71E-03) | 0.09 | 0.36 |
| Medial lemniscus R | 9.87E-03(-1.32E-02 to 3.30E-02) | 0.40 | 0.51 |
| Medial lemniscus L | -3.01E-03(-2.26E-02 to 1.66E-02) | 0.76 | 0.81 |
| Inferior cerebellar peduncle R | -5.45E-03(-2.07E-02 to 9.78E-03) | 0.48 | 0.59 |
| Inferior cerebellar peduncle L | -8.58E-03(-2.40E-02 to 6.80E-03) | 0.27 | 0.52 |
| Superior cerebellar peduncle R | -6.32E-03(-1.87E-02 to 6.06E-03) | 0.31 | 0.52 |
| Superior cerebellar peduncle L | -3.83E-03(-1.64E-02 to 8.75E-03) | 0.54 | 0.65 |
| Cerebral peduncle R | -1.96E-03(-1.38E-02 to 9.89E-03) | 0.74 | 0.81 |
| Cerebral peduncle L | -5.36E-04(-1.32E-02 to 1.22E-02) | 0.93 | 0.95 |
| Anterior limb of internal capsule R | -4.76E-03(-1.47E-02 to 5.15E-03) | 0.34 | 0.50 |
| Anterior limb of internal capsule L | -1.47E-02(-2.75E-02 to -2.02E-03) | **0.02** | 0.19 |
| Posterior limb of internal capsule R | -1.26E-02(-2.53E-02 to 2.56E-05) | 0.05 | 0.30 |
| Posterior limb of internal capsule L | -1.44E-02(-2.80E-02 to -8.42E-04) | **0.04** | 0.26 |
| Retrolenticular part of internal capsule R | -8.25E-03(-2.21E-02 to 5.60E-03) | 0.24 | 0.50 |
| Retrolenticular part of internal capsule L | -7.81E-03(-2.26E-02 to 7.01E-03) | 0.30 | 0.53 |
| Anterior corona radiata R | -8.63E-03(-1.90E-02 to 1.71E-03) | 0.10 | 0.32 |
| Anterior corona radiata L | -5.47E-03(-1.53E-02 to 4.41E-03) | 0.27 | 0.50 |
| Superior corona radiata R | -1.64E-02(-2.74E-02 to -5.39E-03) | **0.004** | 0.20 |
| Superior corona radiata L | -1.21E-02(-2.43E-02 to 1.92E-04) | 0.05 | 0.26 |
| Posterior corona radiata R | -1.26E-02(-2.30E-02 to -2.21E-03) | **0.02** | 0.22 |
| Posterior corona radiata L | -1.16E-02(-2.46E-02 to 1.34E-03) | 0.08 | 0.34 |
| Posterior thalamic radiation R | -4.61E-03(-1.42E-02 to 4.97E-03) | 0.34 | 0.51 |
| Posterior thalamic radiation L | -9.49E-03(-2.23E-02 to 3.36E-03) | 0.14 | 0.39 |
| Sagittal stratum R | -8.40E-03(-2.07E-02 to 3.85E-03) | 0.18 | 0.40 |
| Sagittal stratum L | -1.02E-02(-2.22E-02 to 1.83E-03) | 0.09 | 0.33 |
| External capsule R | -4.19E-03(-1.32E-02 to 4.85E-03) | 0.36 | 0.51 |
| External capsule L | -3.20E-03(-1.46E-02 to 8.17E-03) | 0.58 | 0.64 |
| Cingulum (cingulate gyrus) R | -3.15E-03(-1.11E-02 to 4.81E-03) | 0.43 | 0.55 |
| Cingulum (cingulate gyrus) L | -7.50E-03(-1.51E-02 to 9.43E-05) | 0.05 | 0.28 |
| Cingulum (hippocampus) R | -4.95E-03(-1.62E-02 to 6.27E-03) | 0.38 | 0.51 |
| Cingulum (hippocampus) L | 3.89E-03(-8.91E-03 to 1.67E-02) | 0.55 | 0.64 |
| Fornix (cres) / Stria terminalis R | -8.72E-03(-1.99E-02 to 2.52E-03) | 0.13 | 0.38 |
| Fornix (cres) / Stria terminalis L | -9.08E-03(-2.09E-02 to 2.71E-03) | 0.13 | 0.36 |
| Superior longitudinal fasciculus R | -4.36E-03(-1.33E-02 to 4.60E-03) | 0.33 | 0.52 |
| Superior longitudinal fasciculus L | -6.86E-03(-1.66E-02 to 2.86E-03) | 0.16 | 0.39 |
| Superior fronto-occipital fasciculus R | -5.71E-03(-1.68E-02 to 5.39E-03) | 0.31 | 0.53 |
| Superior fronto-occipital fasciculus L | -6.94E-03(-2.11E-02 to 7.17E-03) | 0.33 | 0.53 |
| Uncinate fasciculus R | -1.09E-04(-1.45E-02 to 1.43E-02) | 0.99 | 0.99 |
| Uncinate fasciculus L | -9.63E-03(-2.31E-02 to 3.83E-03) | 0.16 | 0.40 |
| Tapetum R | -1.91E-02(-3.26E-02 to -5.59E-03) | **0.01** | 0.10 |
| Tapetum L | -1.07E-02(-2.95E-02 to 8.09E-03) | 0.26 | 0.52 |

Note- Coefficients of regression, upper and lower bounds of a 95% confidence interval, and p-values are provided for regressions performed in each WM tract. Significant (<0.05) p-values are in bold.

Supplemental Table 2. Tract-specific linear regression analysis of the association of delivery method with mean diffusivity values after controlling for gestational age at birth, gestational age at scan, 5-minute APGAR, birth weight Z-score, presence of preeclampsia, and presence of chorioamnionitis as covariates.

| **Tract** | **Coefficient (95% Confidence Interval)** | **Original p-value** | **Corrected p-value** |
| --- | --- | --- | --- |
| Middle cerebellar peduncle | 2.83E-05(-4.82E-05 to 1.05E-04) | 0.46 | 0.54 |
| Pontine crossing tract | 5.25E-05(-1.31E-05 to 1.18E-04) | 0.11 | 0.20 |
| Genu of corpus callosum | 2.67E-05(-4.42E-06 to 5.78E-05) | 0.09 | 0.18 |
| Body of corpus callosum | 5.44E-05(1.86E-05 to 9.03E-05) | **0.004** | 0.08 |
| Splenium of corpus callosum | 6.21E-05(1.05E-05 to 1.14E-04) | **0.02** | 0.13 |
| Fornix | 7.95E-05(-6.16E-05 to 2.21E-04) | 0.26 | 0.33 |
| Corticospinal tract R | -4.61E-06(-1.61E-04 to 1.51E-04) | 0.95 | 0.95 |
| Corticospinal tract L | 3.70E-05(-1.21E-04 to 1.95E-04) | 0.64 | 0.71 |
| Medial lemniscus R | 9.02E-05(-5.65E-05 to 2.37E-04) | 0.22 | 0.30 |
| Medial lemniscus L | 4.21E-05(-9.76E-05 to 1.82E-04) | 0.55 | 0.63 |
| Inferior cerebellar peduncle R | 1.14E-04(2.11E-05 to 2.06E-04) | **0.02** | 0.16 |
| Inferior cerebellar peduncle L | 1.08E-04(-1.20E-05 to 2.29E-04) | 0.08 | 0.22 |
| Superior cerebellar peduncle R | 9.43E-05(1.02E-05 to 1.78E-04) | **0.03** | 0.14 |
| Superior cerebellar peduncle L | 9.83E-05(1.64E-05 to 1.80E-04) | **0.02** | 0.12 |
| Cerebral peduncle R | -2.02E-06(-5.58E-05 to 5.17E-05) | 0.94 | 0.96 |
| Cerebral peduncle L | 3.08E-05(-1.67E-05 to 7.83E-05) | 0.20 | 0.30 |
| Anterior limb of internal capsule R | 2.55E-05(-1.56E-05 to 6.66E-05) | 0.22 | 0.30 |
| Anterior limb of internal capsule L | 4.56E-05(-3.76E-06 to 9.50E-05) | 0.07 | 0.24 |
| Posterior limb of internal capsule R | 2.10E-05(-2.03E-06 to 4.40E-05) | 0.07 | 0.23 |
| Posterior limb of internal capsule L | 2.15E-05(-3.39E-06 to 4.64E-05) | 0.09 | 0.19 |
| Retrolenticular part of internal capsule R | 2.74E-05(-3.47E-06 to 5.82E-05) | 0.08 | 0.22 |
| Retrolenticular part of internal capsule L | 3.65E-05(7.40E-06 to 6.55E-05) | **0.01** | 0.18 |
| Anterior corona radiata R | 4.32E-05(-1.02E-05 to 9.66E-05) | 0.11 | 0.20 |
| Anterior corona radiata L | 5.12E-05(-5.01E-06 to 1.07E-04) | 0.07 | 0.22 |
| Superior corona radiata R | 5.50E-05(8.80E-06 to 1.01E-04) | **0.02** | 0.11 |
| Superior corona radiata L | 6.28E-05(1.30E-05 to 1.13E-04) | **0.01** | 0.23 |
| Posterior corona radiata R | 4.64E-05(-8.22E-06 to 1.01E-04) | 0.09 | 0.17 |
| Posterior corona radiata L | 5.10E-05(-1.37E-05 to 1.16E-04) | 0.12 | 0.20 |
| Posterior thalamic radiation R | 2.46E-05(-1.59E-05 to 6.52E-05) | 0.23 | 0.30 |
| Posterior thalamic radiation L | 4.47E-05(-1.76E-05 to 1.07E-04) | 0.16 | 0.24 |
| Sagittal stratum R | 2.74E-05(-1.55E-05 to 7.03E-05) | 0.21 | 0.29 |
| Sagittal stratum L | 3.38E-05(-1.24E-05 to 8.00E-05) | 0.15 | 0.24 |
| External capsule R | 3.13E-05(-4.51E-06 to 6.71E-05) | 0.09 | 0.21 |
| External capsule L | 2.15E-05(-1.21E-05 to 5.51E-05) | 0.21 | 0.30 |
| Cingulum (cingulate gyrus) R | 2.32E-05(-3.95E-06 to 5.03E-05) | 0.09 | 0.18 |
| Cingulum (cingulate gyrus) L | 2.64E-05(-1.99E-06 to 5.48E-05) | 0.07 | 0.25 |
| Cingulum (hippocampus) R | 2.81E-05(-4.46E-06 to 6.07E-05) | 0.09 | 0.19 |
| Cingulum (hippocampus) L | 1.70E-05(-2.28E-05 to 5.67E-05) | 0.40 | 0.48 |
| Fornix (cres) / Stria terminalis R | 4.88E-05(8.59E-06 to 8.90E-05) | **0.02** | 0.15 |
| Fornix (cres) / Stria terminalis L | 7.82E-05(2.91E-05 to 1.27E-04) | **0.002** | 0.11 |
| Superior longitudinal fasciculus R | 3.78E-05(-1.90E-06 to 7.74E-05) | 0.06 | 0.25 |
| Superior longitudinal fasciculus L | 4.16E-05(-5.56E-06 to 8.88E-05) | 0.08 | 0.21 |
| Superior fronto-occipital fasciculus R | 3.90E-05(-3.04E-05 to 1.08E-04) | 0.27 | 0.33 |
| Superior fronto-occipital fasciculus L | 6.54E-05(-1.83E-06 to 1.33E-04) | 0.06 | 0.25 |
| Uncinate fasciculus R | 1.74E-05(-6.05E-05 to 9.54E-05) | 0.66 | 0.70 |
| Uncinate fasciculus L | 7.60E-05(-1.19E-05 to 1.64E-04) | 0.09 | 0.20 |
| Tapetum R | 2.66E-05(-9.89E-05 to 1.52E-04) | 0.67 | 0.70 |
| Tapetum L | 4.94E-05(-1.65E-04 to 2.64E-04) | 0.65 | 0.71 |

Note- Coefficients of regression, upper and lower bounds of a 95% confidence interval, and p-values are provided for regressions performed in each WM tract. Significant (<0.05) p-values are in bold.

Supplemental Table 3. Tract-specific linear regression analysis of the association of delivery method with radial diffusivity values after controlling for gestational age at birth, gestational age at scan, 5-minute APGAR, birth weight Z-score, presence of preeclampsia, and presence of chorioamnionitis as covariates.

| **Tract** | **Coefficient (95% Confidence Interval)** | **Original p-value** | **Corrected p-value** |
| --- | --- | --- | --- |
| Middle cerebellar peduncle | 2.91E-05(-4.49E-05 to 1.03E-04) | 0.43 | 0.52 |
| Pontine crossing tract | 5.27E-05(-1.10E-05 to 1.16E-04) | 0.10 | 0.18 |
| Genu of corpus callosum | 3.51E-05(1.79E-06 to 6.84E-05) | **0.04** | 0.14 |
| Body of corpus callosum | 6.43E-05(2.60E-05 to 1.02E-04) | **0.001** | 0.06 |
| Splenium of corpus callosum | 6.42E-05(1.29E-05 to 1.16E-04) | **0.02** | 0.12 |
| Fornix | 8.78E-05(-5.09E-05 to 2.26E-04) | 0.21 | 0.28 |
| Corticospinal tract R | -7.87E-06(-1.56E-04 to 1.41E-04) | 0.92 | 0.94 |
| Corticospinal tract L | 4.13E-05(-1.07E-04 to 1.90E-04) | 0.58 | 0.63 |
| Medial lemniscus R | 7.80E-05(-6.58E-05 to 2.22E-04) | 0.28 | 0.35 |
| Medial lemniscus L | 4.06E-05(-9.52E-05 to 1.76E-04) | 0.55 | 0.62 |
| Inferior cerebellar peduncle R | 1.11E-04(2.21E-05 to 2.00E-04) | **0.02** | 0.10 |
| Inferior cerebellar peduncle L | 1.07E-04(-5.55E-06 to 2.20E-04) | 0.06 | 0.17 |
| Superior cerebellar peduncle R | 9.13E-05(1.24E-05 to 1.70E-04) | **0.02** | 0.13 |
| Superior cerebellar peduncle L | 9.39E-05(1.71E-05 to 1.71E-04) | **0.02** | 0.10 |
| Cerebral peduncle R | 5.79E-07(-5.09E-05 to 5.21E-05) | 0.98 | 0.98 |
| Cerebral peduncle L | 2.79E-05(-1.94E-05 to 7.52E-05) | 0.24 | 0.31 |
| Anterior limb of internal capsule R | 2.66E-05(-1.48E-05 to 6.79E-05) | 0.20 | 0.29 |
| Anterior limb of internal capsule L | 5.07E-05(3.35E-06 to 9.81E-05) | **0.04** | 0.16 |
| Posterior limb of internal capsule R | 2.68E-05(8.90E-07 to 5.27E-05) | **0.04** | 0.14 |
| Posterior limb of internal capsule L | 2.80E-05(8.19E-07 to 5.51E-05) | **0.04** | 0.13 |
| Retrolenticular part of internal capsule R | 2.77E-05(-6.15E-06 to 6.16E-05) | 0.11 | 0.18 |
| Retrolenticular part of internal capsule L | 3.47E-05(1.37E-06 to 6.81E-05) | **0.04** | 0.14 |
| Anterior corona radiata R | 4.73E-05(-8.81E-06 to 1.03E-04) | 0.10 | 0.18 |
| Anterior corona radiata L | 5.52E-05(-4.78E-06 to 1.15E-04) | 0.07 | 0.17 |
| Superior corona radiata R | 6.25E-05(1.44E-05 to 1.11E-04) | **0.01** | 0.19 |
| Superior corona radiata L | 6.57E-05(1.40E-05 to 1.17E-04) | **0.01** | 0.16 |
| Posterior corona radiata R | 5.23E-05(-3.97E-06 to 1.09E-04) | 0.07 | 0.17 |
| Posterior corona radiata L | 5.49E-05(-1.22E-05 to 1.22E-04) | 0.11 | 0.18 |
| Posterior thalamic radiation R | 2.52E-05(-1.28E-05 to 6.32E-05) | 0.19 | 0.28 |
| Posterior thalamic radiation L | 4.71E-05(-1.22E-05 to 1.06E-04) | 0.12 | 0.19 |
| Sagittal stratum R | 3.21E-05(-1.41E-05 to 7.83E-05) | 0.17 | 0.25 |
| Sagittal stratum L | 3.84E-05(-1.02E-05 to 8.69E-05) | 0.12 | 0.18 |
| External capsule R | 3.11E-05(-3.70E-06 to 6.59E-05) | 0.08 | 0.16 |
| External capsule L | 2.27E-05(-1.28E-05 to 5.82E-05) | 0.21 | 0.28 |
| Cingulum (cingulate gyrus) R | 2.36E-05(-2.55E-06 to 4.98E-05) | 0.08 | 0.17 |
| Cingulum (cingulate gyrus) L | 3.00E-05(3.82E-06 to 5.63E-05) | **0.03** | 0.12 |
| Cingulum (hippocampus) R | 2.95E-05(-2.96E-06 to 6.19E-05) | 0.07 | 0.17 |
| Cingulum (hippocampus) L | 1.32E-05(-2.90E-05 to 5.54E-05) | 0.53 | 0.61 |
| Fornix (cres) / Stria terminalis R | 4.96E-05(1.01E-05 to 8.92E-05) | **0.01** | 0.14 |
| Fornix (cres) / Stria terminalis L | 7.65E-05(2.69E-05 to 1.26E-04) | **0.003** | 0.07 |
| Superior longitudinal fasciculus R | 3.82E-05(-2.23E-06 to 7.87E-05) | 0.06 | 0.17 |
| Superior longitudinal fasciculus L | 4.28E-05(-5.77E-06 to 9.14E-05) | 0.08 | 0.16 |
| Superior fronto-occipital fasciculus R | 3.92E-05(-2.45E-05 to 1.03E-04) | 0.22 | 0.29 |
| Superior fronto-occipital fasciculus L | 6.50E-05(3.63E-06 to 1.26E-04) | **0.04** | 0.15 |
| Uncinate fasciculus R | 1.74E-05(-6.17E-05 to 9.65E-05) | 0.66 | 0.69 |
| Uncinate fasciculus L | 7.70E-05(-1.03E-05 to 1.64E-04) | 0.08 | 0.17 |
| Tapetum R | 4.28E-05(-7.71E-05 to 1.63E-04) | 0.48 | 0.56 |
| Tapetum L | 5.95E-05(-1.57E-04 to 2.76E-04) | 0.58 | 0.62 |

Note- Coefficients of regression, upper and lower bounds of a 95% confidence interval, and p-values are provided for regressions performed in each WM tract. Significant (<0.05) p-values are in bold.

Supplemental Table 4. Tract-specific linear regression analysis of the association of delivery method with fractional anisotropy values in sub-cohort excluding infants born small for gestational age after controlling for gestational age at birth, gestational age at scan, 5-minute APGAR, birth weight Z-score, presence of preeclampsia, and presence of chorioamnionitis as covariates.

| **Tract** | **Coefficient (95% Confidence Interval)** | **Original p-value** | **Corrected p-value** |
| --- | --- | --- | --- |
| Middle cerebellar peduncle | -3.45E-03(-1.48E-02 to 7.94E-03) | 0.55 | 0.75 |
| Pontine crossing tract | -1.95E-03(-1.92E-02 to 1.53E-02) | 0.82 | 0.92 |
| Genu of corpus callosum | -1.11E-02(-2.14E-02 to -7.03E-04) | **0.04** | 0.44 |
| Body of corpus callosum | -1.50E-02(-2.71E-02 to -2.90E-03) | **0.02** | 0.77 |
| Splenium of corpus callosum | -7.88E-03(-1.99E-02 to 4.18E-03) | 0.20 | 0.55 |
| Fornix | -5.54E-03(-2.59E-02 to 1.48E-02) | 0.59 | 0.72 |
| Corticospinal tract R | -1.48E-03(-1.56E-02 to 1.27E-02) | 0.83 | 0.91 |
| Corticospinal tract L | -4.91E-03(-1.84E-02 to 8.54E-03) | 0.47 | 0.75 |
| Medial lemniscus R | 2.47E-03(-2.23E-02 to 2.72E-02) | 0.84 | 0.90 |
| Medial lemniscus L | -1.39E-03(-2.46E-02 to 2.19E-02) | 0.90 | 0.92 |
| Inferior cerebellar peduncle R | -5.85E-03(-2.32E-02 to 1.15E-02) | 0.50 | 0.75 |
| Inferior cerebellar peduncle L | -8.29E-03(-2.51E-02 to 8.54E-03) | 0.33 | 0.68 |
| Superior cerebellar peduncle R | -3.79E-03(-1.68E-02 to 9.21E-03) | 0.56 | 0.73 |
| Superior cerebellar peduncle L | -5.43E-03(-1.79E-02 to 7.00E-03) | 0.38 | 0.71 |
| Cerebral peduncle R | 2.78E-03(-7.96E-03 to 1.35E-02) | 0.61 | 0.71 |
| Cerebral peduncle L | 3.07E-03(-8.92E-03 to 1.51E-02) | 0.61 | 0.70 |
| Anterior limb of internal capsule R | -3.26E-03(-1.41E-02 to 7.63E-03) | 0.55 | 0.73 |
| Anterior limb of internal capsule L | -1.10E-02(-2.41E-02 to 2.06E-03) | 0.10 | 0.52 |
| Posterior limb of internal capsule R | -7.61E-03(-1.81E-02 to 2.84E-03) | 0.15 | 0.65 |
| Posterior limb of internal capsule L | -9.72E-03(-2.36E-02 to 4.11E-03) | 0.16 | 0.66 |
| Retrolenticular part of internal capsule R | -5.86E-03(-2.06E-02 to 8.84E-03) | 0.43 | 0.76 |
| Retrolenticular part of internal capsule L | -5.59E-03(-2.11E-02 to 9.91E-03) | 0.47 | 0.73 |
| Anterior corona radiata R | -9.67E-03(-2.02E-02 to 8.55E-04) | 0.07 | 0.49 |
| Anterior corona radiata L | -7.24E-03(-1.78E-02 to 3.33E-03) | 0.17 | 0.60 |
| Superior corona radiata R | -1.35E-02(-2.51E-02 to -1.92E-03) | **0.02** | 0.56 |
| Superior corona radiata L | -1.28E-02(-2.60E-02 to 4.40E-04) | 0.06 | 0.46 |
| Posterior corona radiata R | -1.21E-02(-2.32E-02 to -9.70E-04) | **0.03** | 0.54 |
| Posterior corona radiata L | -1.26E-02(-2.75E-02 to 2.35E-03) | 0.10 | 0.47 |
| Posterior thalamic radiation R | -5.13E-04(-9.65E-03 to 8.63E-03) | 0.91 | 0.91 |
| Posterior thalamic radiation L | -4.70E-03(-1.70E-02 to 7.59E-03) | 0.45 | 0.77 |
| Sagittal stratum R | -3.45E-03(-1.66E-02 to 9.68E-03) | 0.60 | 0.72 |
| Sagittal stratum L | -7.59E-03(-2.03E-02 to 5.16E-03) | 0.24 | 0.63 |
| External capsule R | -3.72E-03(-1.35E-02 to 6.04E-03) | 0.45 | 0.74 |
| External capsule L | -3.75E-03(-1.58E-02 to 8.31E-03) | 0.54 | 0.76 |
| Cingulum (cingulate gyrus) R | -4.56E-03(-1.25E-02 to 3.39E-03) | 0.26 | 0.64 |
| Cingulum (cingulate gyrus) L | -8.27E-03(-1.74E-02 to 8.34E-04) | 0.07 | 0.44 |
| Cingulum (hippocampus) R | -6.70E-03(-1.90E-02 to 5.63E-03) | 0.28 | 0.64 |
| Cingulum (hippocampus) L | 3.67E-03(-9.59E-03 to 1.69E-02) | 0.58 | 0.73 |
| Fornix (cres) / Stria terminalis R | -8.63E-03(-2.12E-02 to 3.97E-03) | 0.18 | 0.56 |
| Fornix (cres) / Stria terminalis L | -6.67E-03(-1.90E-02 to 5.68E-03) | 0.28 | 0.62 |
| Superior longitudinal fasciculus R | -4.62E-03(-1.42E-02 to 4.97E-03) | 0.34 | 0.68 |
| Superior longitudinal fasciculus L | -5.63E-03(-1.60E-02 to 4.71E-03) | 0.28 | 0.67 |
| Superior fronto-occipital fasciculus R | -8.47E-03(-2.05E-02 to 3.60E-03) | 0.17 | 0.61 |
| Superior fronto-occipital fasciculus L | -3.92E-03(-1.58E-02 to 8.00E-03) | 0.51 | 0.75 |
| Uncinate fasciculus R | 1.20E-03(-1.40E-02 to 1.64E-02) | 0.88 | 0.91 |
| Uncinate fasciculus L | -9.13E-03(-2.26E-02 to 4.30E-03) | 0.18 | 0.54 |
| Tapetum R | -1.44E-02(-2.85E-02 to -2.82E-04) | **0.046** | 0.44 |
| Tapetum L | -8.92E-03(-2.90E-02 to 1.11E-02) | 0.38 | 0.72 |

Note- Coefficients of regression, upper and lower bounds of a 95% confidence interval, and p-values are provided for regressions performed in each WM tract. Significant (<0.05) p-values are in bold.

Supplemental Table 5. Tract-specific linear regression analysis of the association of delivery method with mean diffusivity values in sub-cohort excluding infants born small for gestational age after controlling for gestational age at birth, gestational age at scan, 5-minute APGAR, birth weight Z-score, presence of preeclampsia, and presence of chorioamnionitis as covariates.

| **Tract** | **Coefficient (95% Confidence Interval)** | **Original p-value** | **Corrected p-value** |
| --- | --- | --- | --- |
| Middle cerebellar peduncle | 2.07E-05(-5.06E-05 to 9.21E-05) | 0.56 | 0.64 |
| Pontine crossing tract | 4.64E-05(3.52E-06 to 8.94E-05) | **0.03** | 0.33 |
| Genu of corpus callosum | 1.91E-05(-1.33E-05 to 5.16E-05) | 0.24 | 0.41 |
| Body of corpus callosum | 3.74E-05(-1.24E-06 to 7.60E-05) | 0.06 | 0.28 |
| Splenium of corpus callosum | 5.59E-05(-2.10E-06 to 1.14E-04) | 0.06 | 0.26 |
| Fornix | 5.36E-05(-9.85E-05 to 2.06E-04) | 0.48 | 0.61 |
| Corticospinal tract R | -1.09E-05(-1.69E-04 to 1.47E-04) | 0.89 | 0.89 |
| Corticospinal tract L | 5.45E-05(-1.06E-04 to 2.15E-04) | 0.50 | 0.61 |
| Medial lemniscus R | 9.17E-05(-4.45E-05 to 2.28E-04) | 0.18 | 0.38 |
| Medial lemniscus L | -3.21E-05(-1.93E-04 to 1.28E-04) | 0.69 | 0.72 |
| Inferior cerebellar peduncle R | 5.66E-05(9.31E-06 to 1.04E-04) | **0.02** | 0.48 |
| Inferior cerebellar peduncle L | 2.57E-05(-1.81E-05 to 6.94E-05) | 0.24 | 0.40 |
| Superior cerebellar peduncle R | 4.36E-05(-1.65E-05 to 1.04E-04) | 0.15 | 0.33 |
| Superior cerebellar peduncle L | 6.20E-05(2.57E-06 to 1.21E-04) | **0.04** | 0.25 |
| Cerebral peduncle R | -1.22E-05(-6.92E-05 to 4.48E-05) | 0.67 | 0.73 |
| Cerebral peduncle L | 1.79E-05(-3.68E-05 to 7.27E-05) | 0.51 | 0.62 |
| Anterior limb of internal capsule R | 1.04E-05(-1.30E-05 to 3.37E-05) | 0.38 | 0.53 |
| Anterior limb of internal capsule L | 2.42E-05(-6.99E-06 to 5.55E-05) | 0.13 | 0.29 |
| Posterior limb of internal capsule R | 1.09E-05(-7.10E-06 to 2.90E-05) | 0.23 | 0.42 |
| Posterior limb of internal capsule L | 1.27E-05(-9.56E-06 to 3.51E-05) | 0.26 | 0.40 |
| Retrolenticular part of internal capsule R | 2.53E-05(-4.85E-06 to 5.55E-05) | 0.10 | 0.28 |
| Retrolenticular part of internal capsule L | 3.53E-05(3.50E-06 to 6.71E-05) | **0.03** | 0.48 |
| Anterior corona radiata R | 4.49E-05(-6.70E-06 to 9.65E-05) | 0.09 | 0.28 |
| Anterior corona radiata L | 5.64E-05(-4.16E-08 to 1.13E-04) | 0.05 | 0.27 |
| Superior corona radiata R | 4.44E-05(-3.39E-06 to 9.22E-05) | 0.07 | 0.27 |
| Superior corona radiata L | 4.30E-05(-1.11E-05 to 9.71E-05) | 0.12 | 0.30 |
| Posterior corona radiata R | 5.40E-05(3.99E-06 to 1.04E-04) | **0.03** | 0.28 |
| Posterior corona radiata L | 6.16E-05(-7.77E-06 to 1.31E-04) | 0.08 | 0.30 |
| Posterior thalamic radiation R | 3.19E-05(-7.53E-06 to 7.14E-05) | 0.11 | 0.29 |
| Posterior thalamic radiation L | 4.70E-05(-1.28E-05 to 1.07E-04) | 0.12 | 0.29 |
| Sagittal stratum R | 2.56E-05(-1.85E-05 to 6.97E-05) | 0.25 | 0.40 |
| Sagittal stratum L | 2.70E-05(-2.06E-05 to 7.46E-05) | 0.26 | 0.39 |
| External capsule R | 1.76E-05(-1.01E-05 to 4.53E-05) | 0.21 | 0.42 |
| External capsule L | 1.98E-05(-1.26E-05 to 5.22E-05) | 0.23 | 0.43 |
| Cingulum (cingulate gyrus) R | 1.01E-05(-1.73E-05 to 3.74E-05) | 0.46 | 0.60 |
| Cingulum (cingulate gyrus) L | 1.25E-05(-1.46E-05 to 3.96E-05) | 0.36 | 0.52 |
| Cingulum (hippocampus) R | 1.47E-05(-1.93E-05 to 4.87E-05) | 0.39 | 0.54 |
| Cingulum (hippocampus) L | 1.09E-05(-2.58E-05 to 4.76E-05) | 0.55 | 0.65 |
| Fornix (cres) / Stria terminalis R | 4.26E-05(3.74E-06 to 8.15E-05) | **0.03** | 0.39 |
| Fornix (cres) / Stria terminalis L | 6.30E-05(1.04E-05 to 1.16E-04) | **0.02** | 0.95 |
| Superior longitudinal fasciculus R | 4.32E-05(3.02E-06 to 8.34E-05) | **0.04** | 0.24 |
| Superior longitudinal fasciculus L | 4.75E-05(-6.74E-06 to 1.02E-04) | 0.08 | 0.29 |
| Superior fronto-occipital fasciculus R | 8.92E-06(-3.39E-05 to 5.17E-05) | 0.68 | 0.72 |
| Superior fronto-occipital fasciculus L | 4.84E-05(-7.69E-06 to 1.05E-04) | 0.09 | 0.27 |
| Uncinate fasciculus R | 6.00E-06(-6.70E-05 to 7.90E-05) | 0.87 | 0.89 |
| Uncinate fasciculus L | 4.90E-05(-3.32E-05 to 1.31E-04) | 0.24 | 0.42 |
| Tapetum R | 4.65E-05(-7.82E-05 to 1.71E-04) | 0.46 | 0.61 |
| Tapetum L | 5.88E-05(-1.49E-04 to 2.66E-04) | 0.57 | 0.64 |

Note- Coefficients of regression, upper and lower bounds of a 95% confidence interval, and p-values are provided for regressions performed in each WM tract. Significant (<0.05) p-values are in bold.

Supplemental Table 6. Tract-specific linear regression analysis of the association of delivery method with radial diffusivity values in sub-cohort excluding infants born small for gestational age after controlling for gestational age at birth, gestational age at scan, 5-minute APGAR, birth weight Z-score, presence of preeclampsia, and presence of chorioamnionitis as covariates.

| **Tract** | **Coefficient (95% Confidence Interval)** | **Original p-value** | **Corrected p-value** |
| --- | --- | --- | --- |
| Middle cerebellar peduncle | 2.06E-05(-4.96E-05 to 9.07E-05) | 0.56 | 0.64 |
| Pontine crossing tract | 4.40E-05(1.31E-06 to 8.67E-05) | **0.04** | 0.26 |
| Genu of corpus callosum | 2.59E-05(-8.99E-06 to 6.07E-05) | 0.14 | 0.31 |
| Body of corpus callosum | 4.79E-05(7.06E-06 to 8.87E-05) | **0.02** | 0.54 |
| Splenium of corpus callosum | 5.71E-05(4.91E-09 to 1.14E-04) | **0.0499** | 0.24 |
| Fornix | 5.58E-05(-9.44E-05 to 2.06E-04) | 0.46 | 0.58 |
| Corticospinal tract R | -1.12E-05(-1.62E-04 to 1.40E-04) | 0.88 | 0.88 |
| Corticospinal tract L | 5.37E-05(-9.76E-05 to 2.05E-04) | 0.48 | 0.59 |
| Medial lemniscus R | 8.51E-05(-4.87E-05 to 2.19E-04) | 0.21 | 0.34 |
| Medial lemniscus L | -2.78E-05(-1.84E-04 to 1.28E-04) | 0.72 | 0.75 |
| Inferior cerebellar peduncle R | 5.85E-05(1.06E-05 to 1.06E-04) | **0.02** | 0.85 |
| Inferior cerebellar peduncle L | 3.05E-05(-1.41E-05 to 7.51E-05) | 0.18 | 0.31 |
| Superior cerebellar peduncle R | 4.20E-05(-1.45E-05 to 9.86E-05) | 0.14 | 0.32 |
| Superior cerebellar peduncle L | 6.03E-05(5.57E-06 to 1.15E-04) | **0.03** | 0.30 |
| Cerebral peduncle R | -1.22E-05(-6.75E-05 to 4.31E-05) | 0.66 | 0.72 |
| Cerebral peduncle L | 1.40E-05(-3.95E-05 to 6.75E-05) | 0.60 | 0.67 |
| Anterior limb of internal capsule R | 1.18E-05(-1.35E-05 to 3.70E-05) | 0.35 | 0.49 |
| Anterior limb of internal capsule L | 2.87E-05(-4.73E-06 to 6.22E-05) | 0.09 | 0.26 |
| Posterior limb of internal capsule R | 1.47E-05(-4.02E-06 to 3.34E-05) | 0.12 | 0.31 |
| Posterior limb of internal capsule L | 1.77E-05(-7.29E-06 to 4.27E-05) | 0.16 | 0.32 |
| Retrolenticular part of internal capsule R | 2.45E-05(-1.04E-05 to 5.94E-05) | 0.17 | 0.32 |
| Retrolenticular part of internal capsule L | 3.24E-05(-4.26E-06 to 6.91E-05) | 0.08 | 0.28 |
| Anterior corona radiata R | 5.02E-05(-5.07E-06 to 1.05E-04) | 0.07 | 0.30 |
| Anterior corona radiata L | 6.16E-05(-1.91E-07 to 1.23E-04) | 0.05 | 0.22 |
| Superior corona radiata R | 5.04E-05(2.93E-07 to 1.01E-04) | **0.049** | 0.26 |
| Superior corona radiata L | 4.87E-05(-7.07E-06 to 1.05E-04) | 0.09 | 0.27 |
| Posterior corona radiata R | 5.88E-05(5.88E-06 to 1.12E-04) | **0.03** | 0.36 |
| Posterior corona radiata L | 6.52E-05(-7.64E-06 to 1.38E-04) | 0.08 | 0.29 |
| Posterior thalamic radiation R | 2.79E-05(-1.01E-05 to 6.60E-05) | 0.15 | 0.31 |
| Posterior thalamic radiation L | 4.52E-05(-1.41E-05 to 1.04E-04) | 0.13 | 0.32 |
| Sagittal stratum R | 2.59E-05(-2.27E-05 to 7.45E-05) | 0.29 | 0.41 |
| Sagittal stratum L | 2.99E-05(-2.03E-05 to 8.01E-05) | 0.24 | 0.36 |
| External capsule R | 1.92E-05(-1.10E-05 to 4.93E-05) | 0.21 | 0.33 |
| External capsule L | 2.20E-05(-1.46E-05 to 5.85E-05) | 0.23 | 0.36 |
| Cingulum (cingulate gyrus) R | 1.21E-05(-1.46E-05 to 3.88E-05) | 0.37 | 0.49 |
| Cingulum (cingulate gyrus) L | 1.75E-05(-8.00E-06 to 4.30E-05) | 0.17 | 0.32 |
| Cingulum (hippocampus) R | 1.87E-05(-1.58E-05 to 5.32E-05) | 0.28 | 0.41 |
| Cingulum (hippocampus) L | 8.36E-06(-3.14E-05 to 4.81E-05) | 0.67 | 0.72 |
| Fornix (cres) / Stria terminalis R | 4.36E-05(3.81E-06 to 8.35E-05) | **0.03** | 0.26 |
| Fornix (cres) / Stria terminalis L | 6.11E-05(8.03E-06 to 1.14E-04) | **0.02** | 0.40 |
| Superior longitudinal fasciculus R | 4.38E-05(2.08E-06 to 8.56E-05) | **0.04** | 0.27 |
| Superior longitudinal fasciculus L | 5.13E-05(-9.92E-06 to 1.12E-04) | 0.10 | 0.26 |
| Superior fronto-occipital fasciculus R | 1.40E-05(-3.07E-05 to 5.87E-05) | 0.53 | 0.64 |
| Superior fronto-occipital fasciculus L | 4.76E-05(-7.23E-06 to 1.02E-04) | 0.09 | 0.26 |
| Uncinate fasciculus R | 5.62E-06(-6.93E-05 to 8.05E-05) | 0.88 | 0.90 |
| Uncinate fasciculus L | 5.13E-05(-2.91E-05 to 1.32E-04) | 0.21 | 0.35 |
| Tapetum R | 5.46E-05(-6.85E-05 to 1.78E-04) | 0.38 | 0.49 |
| Tapetum L | 6.58E-05(-1.46E-04 to 2.78E-04) | 0.54 | 0.63 |

Note- Coefficients of regression, upper and lower bounds of a 95% confidence interval, and p-values are provided for regressions performed in each WM tract. Significant (<0.05) p-values are in bold.

**Supplemental Figures**


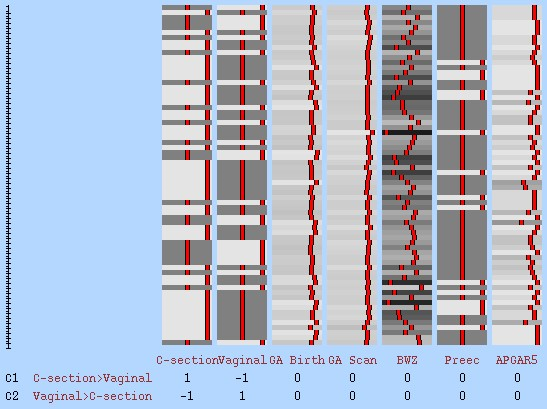


Supplemental Figure 1. Visual representation of the design and contrast files used during the “randomise” operation to perform voxel-wise general linear model analyses. C-section = infants delivered via C-section. Vaginal = infants delivered vaginally. GA birth = gestational age at birth. GA scan = gestational age at scan. BWZ = birth weight Z-score. Preec = presence of preeclampsia. APGAR5 = 5-minute APGAR score.
